# Feeling Younger, Feeling Older: Unpacking the Complexities of Subjective Age

**DOI:** 10.1093/geroni/igaf122.218

**Published:** 2025-12-31

**Authors:** M Clara de Paula Couto, David Weiss

## Abstract

Subjective age, or how young or old individuals feel compared to their chronological age, influences various developmental outcomes. This symposium explores subjective age through five studies using diverse methodologies, including longitudinal and experimental designs, to examine the determinants, consequences, and mechanisms of subjective age. Weiss et al. examine how social and temporal comparisons shape subjective aging. Their findings reveal that social comparisons lead to a relative younger subjective age, while temporal comparisons often result in more negative self-perceptions of aging and an older subjective age. de Paula Couto et al. build on the dual-process theory of developmental regulation. showing that individuals counteract societal negative attitudes towards aging by feeling younger (assimilation) or redefining old age (accommodation). Younger participants felt younger in valued domains, while older participants raised the threshold for old age. Gourley and Chasteen investigate the drawbacks of feeling younger in later adulthood from a social identity threat perspective. They find that a younger subjective age can lead to negative social evaluations from younger and middle-aged adults, thus countering its benefits. Kornadt et al. examine loneliness as a determinant of subjective age in a longitudinal study, showing that greater loneliness leads to an older subjective age trajectory, mediated by stress, highlighting its psychological toll and accelerating effects on subjective aging. Finally, Sabatini et al. explore subjective age’s relation to future care planning, finding that dementia caregivers feel older and are more engaged in care preparation compared to never-caregivers, suggesting that a relative older subjective age fosters proactive future planning.

